# Understanding the Dynamics of Violent Political Revolutions in an Agent-Based Framework

**DOI:** 10.1371/journal.pone.0154175

**Published:** 2016-04-22

**Authors:** Alessandro Moro

**Affiliations:** Department of Economics, Ca’ Foscari University, Venice, Italy; Université Toulouse 1 Capitole, FRANCE

## Abstract

This paper develops an agent-based computational model of violent political revolutions in which a subjugated population of citizens and an armed revolutionary organisation attempt to overthrow a central authority and its loyal forces. The model replicates several patterns of rebellion consistent with major historical revolutions, and provides an explanation for the multiplicity of outcomes that can arise from an uprising. The relevance of the heterogeneity of scenarios predicted by the model can be understood by considering the recent experience of the Arab Spring involving several rebellions that arose in an apparently similar way, but resulted in completely different political outcomes: the successful revolution in Tunisia, the failed protests in Saudi Arabia and Bahrain, and civil war in Syria and Libya.

## Introduction

The phenomenon of political revolutions has once again caught the attention of researchers in the wake of the recent wave of uprisings in the Arab World. The main purpose of this paper is to present an agent-based computational model that outlines the common dynamics of major political revolutions and replicates a number of stylised facts.

The model features three types of agent that interact in a bidimensional torus space: a population of citizens who are oppressed by a central government; members of a revolutionary organisation who attempt to overthrow the government by an armed uprising; and loyal policemen who are tasked with suppressing the rebellion.

This simple model is able to reproduce several patterns of rebellion consistent with major historical revolutions: a pre-revolutionary period characterised by spontaneous riots, motivated mainly by poor economic conditions and social inequality, gives way to an actual revolutionary rebellion, in which organised elements mobilise popular masses against the central government.

Moreover, the model provides an explanation for the multiplicity of outcomes that can arise from an uprising: a completely successful revolution leading to the overthrow of the central authority; a failed rebellion followed by a return to the status quo; an intermediate case where the uprising is unable to change the political system, but is sufficiently strong to destabilise the country and drive it towards anarchy.

The heterogeneity of scenarios predicted by the model is not highlighted in existing literature, and its importance can be understood by considering the recent experience of the Arab Spring involving several rebellions that arose in an apparently similar way, but resulted in completely different political outcomes: e.g. the successful revolution in Tunisia, the failed protests in Saudi Arabia and Bahrain, and civil war in Syria and Libya.

For decades, the most popular conceptualisations of revolution were the Marxian theory and the relative deprivation theory. The former emphasises the role of changes in production methods in generating discontent and rebellion; the latter focuses on the gap between economic expectations and realised economic performances to explain the sense of frustration and, consequently, riot participation. Both theories establish an automatic link between the structural conditions that generate grievance in society and the likelihood of revolutionary episodes. Moreover, in both theories participation in rebellion is motivated by a collective good argument, such as the desire to change the oppressive social order. Two of the most influential scholars in this stream of literature are Skocpol [1] with regard to the Marxian theory and Davies [2] concerning the relative deprivation theory. For a complete review of the political science literature about revolutions, see Goldstone [3].

In contrast, Tullock [4] develops an economic approach to explain participation in revolutions: since the benefit of an extra unit of public good is small relative to the cost of obtaining it by participating in a rebellion, individuals decide whether or not to participate based on their private gains or losses. Silver [5] provides a classification of revolutions based on Tullock’s theory. Moreover, Kuran [6–8] criticises the idea of an automatic relationship between social grievance and revolution, arguing that most historical revolutions were unanticipated. He provides an explanation based on the observation that people who dislike their government tend to conceal their political preferences as long as the opposition seems weak. For this reason, regimes that appear to be absolutely stable may experience a sudden loss of support in the event of a minor increase in the size of the opposition, even if triggered by insignificant events.

The economic and political science literature have endeavoured to solve the collective action problems inherent in revolutions. For example, in criticism of Tullock, Lichbach [9–11] identifies a number of solutions based on sanctioning and group identification methods. These solutions include the possibility of imposing community obligations, establishing institutional mechanisms, arranging contracts and using authority. For an example of an institutional kind of solution in the context of 18th century merchant sailors, see Leeson [12].

Furthermore, in line with Kuran’s theory, Rubin [13] argues that cascades of preference revelation are more likely to occur following a major shock in highly centralised regimes. This is because citizens in such political systems have a greater incentive to conceal their true political opinions in order to avoid economic or legal sanctions being imposed by the central authority. Makowsky and Rubin [14] extend the previous work by developing an agent-based model to study how social network technology favours preference revelation in centralised societies.

A number of game theoretic papers have also been produced that analyse the economic causes of political change: for instance, following Acemoglu and Robinson’s [15] model of the economic origins of democracy, Ellis and Fender [16] derive conditions under which democracy arises peacefully, when it occurs after a revolution, and when oligarchic governments persist. An alternative view is represented by the paper of Gard-Murray and Bar-Yam [17], who argue that democracies are more systemically complex than autocracies and, since violent revolutions are likely to disrupt existing evolved complexity, dictatorships have higher chances of emerging after uprisings.

Finally, this paper is also influenced to a great extent by Granovetter’s [18] theory about threshold models of collective behaviours and by Epstein’s [19] agent-based model of civil violence. According to Granovetter, individuals face many situations with multiple alternatives, and the costs and benefits associated with these alternatives depend on how many other individuals have chosen the various options in the past. For this reason, each individual has a personal threshold, and decides to join collective action, such as a riot or a strike, if the number of people participating at that time exceeds this threshold. Following this idea, Epstein develops an agent-based model of civil violence involving two types of player, agents and cops, interacting in a bidimensional torus space. In this model the agents decide to rebel against the government if their level of grievance corrected by the risk of being arrested by the cops exceeds their activation threshold. One of the main findings of this model is that intermittent outbursts of violence occur, distributed irregularly over time and space. Another study that explores the temporal and spatial diffusion of civil unrest is that produced by Braha [20]. In particular, his paper demonstrates that the distribution of real episodes of civil violence can be replicated using a spatially extended dynamical model that incorporates the effects of social and communication networks.

The rest of the paper is organised as follows: the next section describes the model; in the Results section, the three outcomes generated by the model are presented and their dependence on the model parameters is analysed using graphical and statistical tools; the final section discusses the results and their relevance for analysing contemporary revolutions.

## Methods

In the agent-based computational model presented in this paper, there are three types of agent that interact in a bidimensional torus space: citizens, policemen and revolutionaries. Citizens are members of a population subjugated to a central authority who decide whether or not to rebel against the government based on their degree of economic and political grievance. Revolutionaries are members of an organised opposition group that seeks to overthrow the central government by an armed uprising. Policemen are the forces loyal to the central authority that have been tasked to suppress any kind of revolt by arresting rebellious citizens and killing revolutionaries.

In this section, the features of each agent are described in detail, beginning with the citizen specification. As in Epstein [19], social grievance represents the motivation that potentially leads citizens to revolt; for each citizen *i* the grievance is assumed to be the product of an index of economic hardship *H* and a measure of government illegitimacy, defined as 1 − *l*, where *l* is a parameter measuring the legitimacy of the central authority:

$$\begin{array}{c} G\left(y_{i}\right)=\left(1-l\right)H\left(y_{i}\right) \end{array}$$ (1)

In contrast to Epstein’s specification, the perceived hardship, and consequently grievance, is a function of citizens’ income *y*_*i*_. In fact, each citizen is endowed with an income drawn from a lognormal distribution, whose density function is:

$$\begin{array}{c} d\left(y_{i}\right)=\frac{1}{\sqrt{2\pi }by_{i}}\exp\left[-\frac{{\left(\ln y_{i}-a\right)}^{2}}{2b^{2}}\right],\;y_{i}>0 \end{array}$$ (2)

The functional form chosen for the hardship index is:

$$\begin{array}{c} H\left(y_{i}\right)=\frac{\exp\left[E\left(y_{i}\right)-y_{i}\right]}{1+\exp\left[E\left(y_{i}\right)-y_{i}\right]} \end{array}$$ (3)

This function allows each citizen’s economic condition to be mapped to a value in the (0, 1) interval. This index is a logistic transformation of the difference between citizens’ income and the expected income in the population $E\left(y_{i}\right)=\exp\left(a+\frac{b^{2}}{2}\right)$. Given this monotonic transformation, hardship is a decreasing function of citizens’ income. This expression is similar to the definition of grievance employed by Kim and Hanneman [21]. The main difference with respect to their specification is that the two authors use a local measure of inequality, i.e. the distance between each agent’s wage and the average wage in the agent’s neighbourhood; conversely, in this model a global measure of inequality is preferred.

On the other hand, the cost of participating in a rebellion is defined as the product of the estimated probability of being arrested *A*_*i*_ and the opportunity cost of joining a revolt *J*:

$$\begin{array}{c} N\left(y_{i}\right)=A_{i}J\left(y_{i}\right) \end{array}$$ (4)

In fact, each citizen estimates the probability of being arrested before actively joining a rebellion. This estimated probability is defined as in Epstein [19]: it is an increasing function of the ratio of policemen to already rebellious agents inside the citizen’s vision radius. In particular, in this model rebel agents can either be citizens and revolutionaries:

$$\begin{array}{c} A_{i}=1-\exp\left[-w_{1}\left(\frac{P_{i}^{v}}{1+C_{i}^{v}+R_{i}^{v}}\right)\right] \end{array}$$ (5)

where $P_{i}^{v}$, $C_{i}^{v}$ and $R_{i}^{v}$ represent the number of policemen, rebellious citizens and active revolutionaries within the citizen’s vision, respectively. The vision, a circular neighbourhood with centre located in the citizen’s position and a radius equal to *v*, represents the set of lattice positions probed by the citizen. The one in the previous formula makes explicit that, before participating in a riot, a citizen will count himself as an active agent: thus the ratio is always well defined. In practice, the floor operator is applied to the ratio of policemen to rebel agents, as in Wilensky’s [22] version of Epstein’s model.

If an active citizen is arrested by a policeman, he remains in jail for a number of periods drawn from a uniform distribution on the (0, *j*_*max*_) interval. For this reason, the opportunity cost of rebelling is defined as a function of the maximum number of periods in jail *j*_*max*_, multiplied by income loss whilst in jail:

$$\begin{array}{c} J\left(y_{i}\right)=2\frac{\exp\left[w_{2}\left(y_{i}j_{max}\right)\right]}{1+\exp\left[w_{2}\left(y_{i}j_{max}\right)\right]}-1 \end{array}$$ (6)

Since the inner argument of the logistic transformation is positive, given that income assumes only positive values, the logistic function is rescaled in order to define a cost function *J* on the (0, 1) interval. Expression (6) is also consistent with the literature on political violence, which finds a negative relationship between income and participation in civil violence phenomena. For example, Collier and Hoeffler [23, 24] and Fearon and Laitin [25], using cross-country regressions, find that economic growth and per capita income correlate negatively with the risk of civil conflict. Moreover, Miguel, Satyanath and Sergenti [26] identify a causal negative effect between positive income shocks and civil war incidence in Sub-Saharan African countries employing an instrumental variable approach.

Having defined the incentives and the costs underlying participation in riot activities, it is now possible to specify citizens’ rule of activation. Citizens particularly become active, meaning that they decide to rebel against the government, if the difference between their social grievance and the expected opportunity cost of joining a riot exceeds a fixed threshold; otherwise, they will keep quiet. The citizens’ rule is therefore:

Rule C: if *G*(*y*_*i*_) − *N*(*y*_*i*_) > *f* be active; otherwise, keep quiet.

This inequality can be interpreted using Kuran’s [6] theory: the left-hand side represents the expected utility of expressing opposition to the central authority in public; the right-hand side *f* is the constant utility of keeping quiet and concealing private political preferences.

The revolutionaries’ behaviour is simpler. Revolutionaries are members of an organised group that attempts to overthrow the government by an armed conflict. This kind of agent can be interpreted as a proper revolutionary group or as defected elements from the military that decide to side with the population in revolt. Historical examples of the first type of organisation include the Bourgeois Militia of Paris in the French Revolution (1789); the Bolsheviks and Red Guards in the Russian Revolution (1917); the leftist revolutionaries of the Organisation of Iranian People’s Fedai Guerrillas in the Iranian Revolution (1979); the Muslim Brotherhood in the Egyptian Revolution (2011); the jihadist group of the Islamic State of Iraq and the Levant in the Syrian Civil War (2011), and many others. Defections from the military are also very common in all revolutions: a typical example is the pro-Khomeini members of the Iranian Air Force who fought against the loyal Immortal Guards during the 1979 uprisings.

It is assumed that revolutionaries behave according to the following rule:

Rule R: if $\frac{R+C}{P}>n$ be active and kill a randomly selected policeman inside vision radius *v* with a probability equal to *r*; otherwise, remain hidden.

Here *R*, *C* and *P* are the total number of revolutionaries, the total number of active citizens and the total number of policemen, respectively. Rule R means that revolutionaries decide to become active when the ratio of rebel forces to policemen loyal to the government exceeds a given threshold *n*. In this respect, revolutionaries are different from citizens: citizens choose how to behave according to local information available within their vision radius. In contrast, revolutionaries act on the basis of global information and decide when to start a revolution by employing a threshold-based rule involving the total number of active citizens in the population. In fact, it is assumed that the revolutionary organisation is spread across the country, enabling it to obtain an estimate of the total number of active agents in the population.

When a revolutionary is active, he kills a randomly selected policeman in his vision radius with a probability equal to *r*. Otherwise, when the ratio is less than the fixed threshold, all revolutionaries will remain hidden among quiet citizens and policemen will be unable to identify them.

As far as policemen are concerned, they simply inspect the lattice positions inside their vision radius and randomly choose an active citizen or active revolutionary: if the randomly selected agent is a citizen, the policeman will arrest him, or will kill him if he is an active revolutionary with a probability equal to *p*. The policemen’s rule is therefore:

Rule P: randomly select an agent from the active citizens and active revolutionaries within vision radius *v*. If the randomly selected agent is a citizen, arrest him; if he is a revolutionary, kill him with a probability equal to *p*.

The same vision radius *v* is assumed for citizens, revolutionaries and policemen. Furthermore, parameters *r* and *p* can also be interpreted in terms of weapon precision or, more broadly, in terms of effectiveness in the military capacity of the conflicting parties. Once killed, revolutionaries and policemen are simply removed from the bidimensional space.

Finally, citizens who are not in jail, revolutionaries and policemen who are not killed can move in the lattice space to a random site without agents or in which there are only jailed citizens following this simple rule:

Rule M: within vision radius *v*, randomly move to an empty site or to a site in which there are only jailed citizens.

Table 1 presents the parameter values that are kept constant in all model simulations: the values assigned to the lognormal parameters (*a*, *b*) and the cost function parameter *w*_2_ are selected in order to obtain a widespread distribution of hardship and opportunity costs on the (0, 1) interval, avoiding concentration at the extremes of that interval; the other values are assigned according to those selected by Epstein [19]. The next section of the paper investigates the effects of the new parameters introduced by the present model, i.e. the military effectiveness of the two factions (*p*, *r*) and the revolutionaries’ threshold *n*.

**Table 1:** Parameter values fixed in all model simulations.

Parameter	Description	Value
*d*_*C*_	Citizen density	70%
*d*_*R*_	Revolutionary density	3%
*d*_*P*_	Policeman density	4%
*l*	Government legitimacy	0.85
(*a*, *b*)	Lognormal parameters	(0.5, 0.5)
*w*_1_	Arrest probability parameter	2.3
*w*_2_	Cost function parameter	0.025
*f*	Citizens’ activation threshold	0.1
*v*	Vision radius	7
*j*_*max*_	Maximum number of periods in jail	30
Lattice Dimensions	Dimensions of the bidimensional space	40 × 40

At the beginning of each model simulation, the random values *y*_*i*_ are drawn from the lognormal distribution and the different agents are randomly situated on the sites of the lattice. Then, an agent is selected at random. Under rule M, he moves to a random position within his vision, where he acts according to rule C if he is a citizen, rule R if he is a revolutionary or rule P if he is a policeman. This procedure is replicated until a given time or a specific condition (e.g. all revolutionaries or policemen are killed) is reached. The model was written using NetLogo (Wilensky [27]), whereas the statistical analysis was performed using R (R Core Team [28]): details of implementation and the code are in the S1 Code file, in the supplementary material.

## Results

### Model Outcomes

Three distinct outcomes can be identified by simulating this simple model: a successful revolution in which all policemen are killed by revolutionaries, leading to an overthrow of the central government; a failed revolution followed by a state of anarchy due to the large number of policemen killed; a completely failed revolution with only a few policemen killed, signifying a return to the status quo after the uprising.

**Fig 1:**
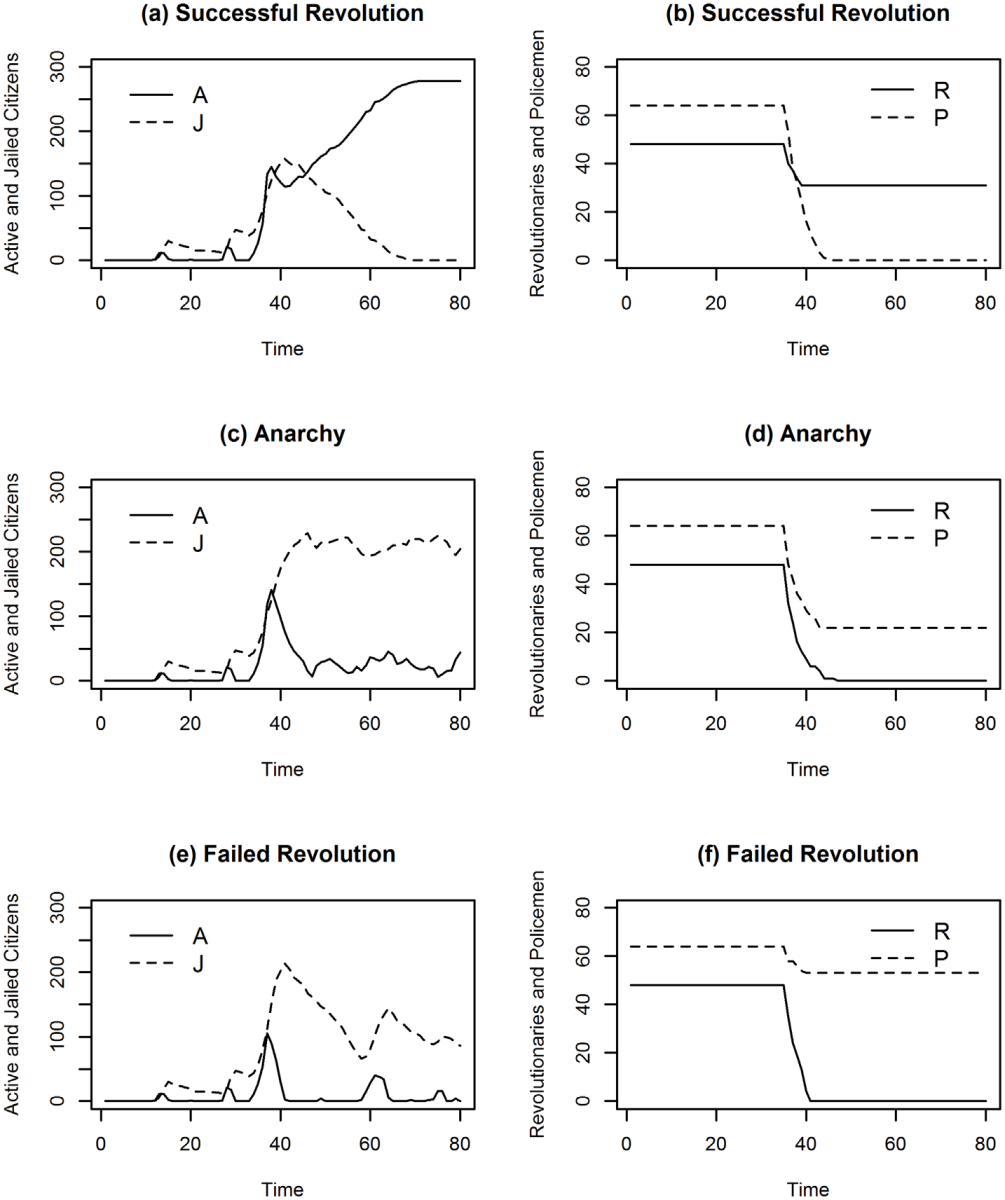
The three model outcomes. Time series graph for the different model scenarios: (a) time series of the number of active and jailed citizens in a successful revolution; (b) time series of the number of revolutionaries and policemen who have survived a successful revolution; (c) time series of the number of active and jailed citizens in an anarchic scenario; (d) time series of the number of revolutionaries and policemen who have survived an anarchic scenario; (e) time series of the number of active and jailed citizens in a failed revolution; (f) time series of the number of revolutionaries and policemen who have survived a failed revolution.

Fig 1 shows these possible outcomes with three simulations in which the random seed and the value of *n* (*n* = 1.2) are the same but the two precision parameters take different values: in particular, in the two upper graphs *p* = 0.4 and *r* = 0.3; in the middle pictures *p* = 0.9 and *r* = 0.3; finally, in the lower graphs *p* = 0.9 and *r* = 0.1. All three simulations start with a period of instability characterised by minor revolts where the poorest component of the population, made up of citizens with the greatest degree of grievance and the lowest opportunity cost, decides to rebel. However, these riots are too small, meaning that they do not degenerate into a revolution. This politically unstable pre-revolutionary period is a common feature of many historical revolutions: e.g. the strikes and workers’ demonstrations in Russia (1917), Iran (1977–1978) and the Arab World (2011), motivated to a great extent by poor economic conditions such as low wages, high inflation (especially high food prices, as documented by Lagi, Bertrand and Bar-Yam [29]), inequality, unemployment, as well as by a small degree of political legitimacy, due to the Russian Tsar’s war defeat or the Shah’s unpopular westernised costumes in the case of Iran.

Around time 30 a major riot occurs, and the revolutionaries’ threshold rule is satisfied: this implies that revolutionaries become active and the rebellion, that started as a riot motivated by the poorest citizens’ bad economic conditions, has now the features of a political revolution. The revolutionaries’ threshold *n* therefore plays an important role because it determines the time at which revolutionaries will become active (in this specific simulation, *n* = 1.2). When revolutionaries become active, the citizens’ estimated probability of arrest is lowered, creating a surge in the number of active citizens. Moreover, this effect is reinforced by the fact that revolutionaries start killing policemen, again lowering the probability of arrest. What happens next depends on the parameters that regulate the relative strength of the two factions.

In the two upper graphs (*p* = 0.4 and *r* = 0.3), once revolutionaries have taken action, a large number of citizens become active, and policemen find more readily active citizens than revolutionaries: this explains why, following the surge, many citizens are arrested and only a few revolutionaries are killed. Hidden among active citizens, revolutionaries shoot policemen; when many are killed, the number of active citizens starts to increase again and, when all policemen have been killed, it reaches its maximum, i.e. all citizens with a degree of grievance exceeding the threshold become active: the revolution is complete and the government is overthrown. Political scientists (see Goldstone [3]) have observed a common feature in all successful revolutions: they only occur when there is a link between mass mobilisation and the revolutionary movements that place themselves at the head of popular revolts, giving them organisation and coherence. This occurred with the Bolsheviks and the workers’ riots in 1917 and with the Ayatollah Khomeini and the protests in the Iran’s bazaars. The model is capable of capturing this link between popular spontaneous riots and organised action by revolutionaries. Examples of successful rebellions are represented by the three major historical revolutions in France (1789), Russia (1917) and Iran (1979), as well as by the recent uprisings in Tunisia (2011). In all these cases, the pre-revolutionary government is overthrown and a new order is established. S1 Video presents the evolution of a successful revolution showing the bidimensional space and the interactions between different agents.

Conversely, in the middle graphs (*p* = 0.9 and *r* = 0.3), after the surge of active citizens, the armed conflict between revolutionaries and policemen is won by the latter. Nevertheless, a large number of policemen are killed and the revolution is followed by a period of major, never-ending turmoil: the huge reduction in the state’s legal capacity, caused by the uprising, drives the country towards anarchy. A similar anarchic post-revolutionary situation usually follows a rebellion when the percentage of policemen killed exceeds 40% in the simulations. The anarchic outcome resembles the present civil war scenarios in Syria and Libya, where the 2011 insurrections completely destabilised these countries, reducing their government’s capacity to rule. S2 Video shows the emergence of an anarchic outcome after an uprising.

Finally, in the lower graphs (*p* = 0.9 and *r* = 0.1), the difference in the military effectiveness of the two factions is too large, and only a few policemen are killed during the uprising (usually less than 40%). This means that, following a major rebellion, the situation is similar to that in the pre-revolutionary period: the status quo is maintained. Here the analogy is with the 2011 riots in Saudi Arabia and Bahrain, where opposition groups were very weak from a military perspective, and only a few police officers were killed in the street violence episodes. A simulated example of a failed revolution is presented in S3 Video.

In order to explore how the different outcomes of the model vary with the parameters associated with policemen’s and revolutionaries’ precision as well as with the threshold revolutionaries employ in their decision rule, the model was simulated for different values of these parameters: in particular, *n* takes values in the set {0.7, 0.8, 0.9, 1.0, 1.1, 1.2, 1.3, 1.4}, whereas the two precision parameters *p* and *r* assume values in {0.1, 0.2, …, 0.8, 0.9} and {0.1, 0.2, 0.3, 0.4}, respectively. Finally, for each combination of these parameter values, the model is simulated 60 times, for a total of 17,280 simulations (S1 Dataset file contains all of the simulations performed). Each simulation is halted after 300 time steps.

**Fig 2:**
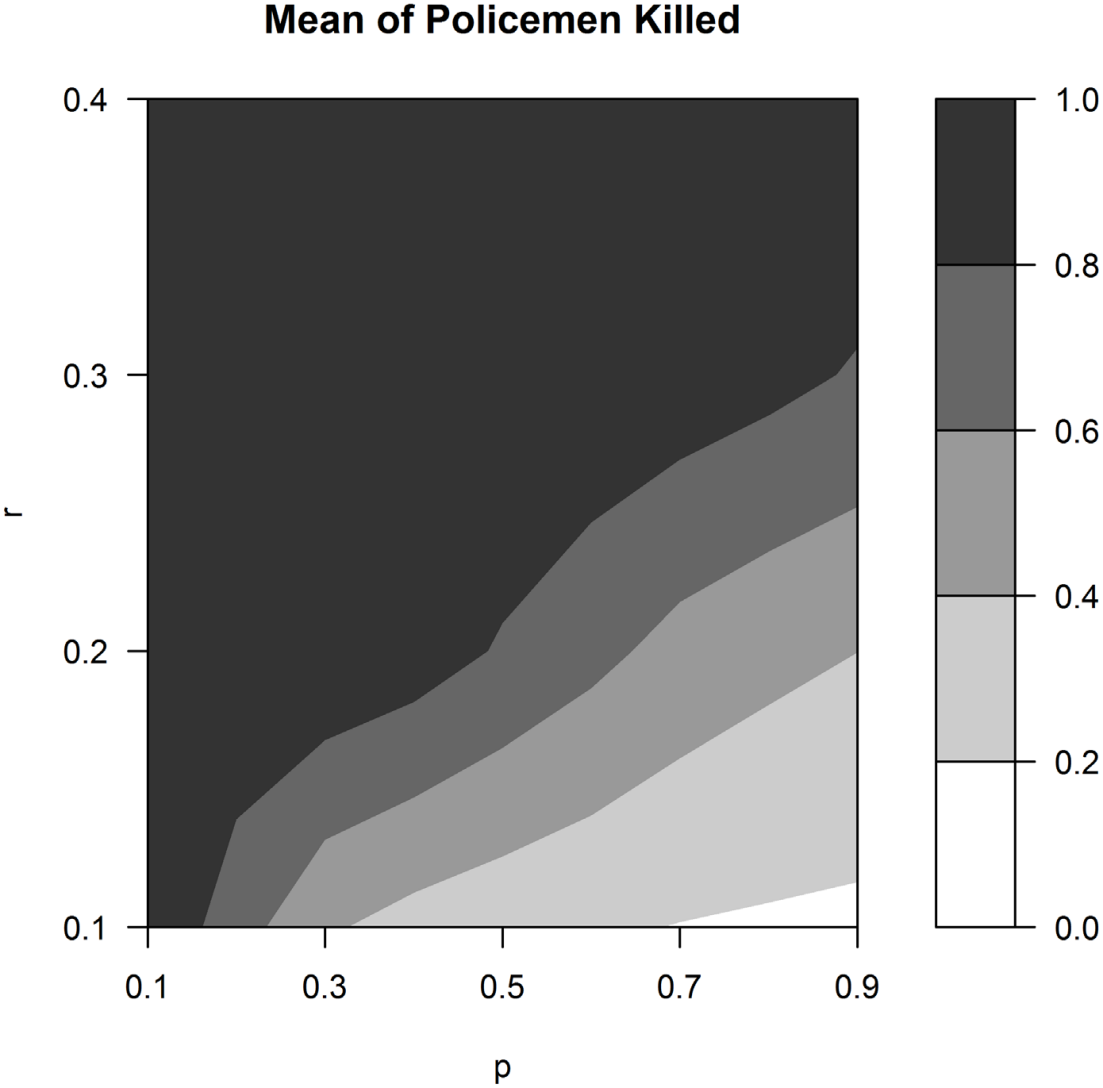
Average proportion of policemen killed for different values of the two precision parameters. For each combination of *p* and *r*, the average proportion of policemen killed is calculated employing 480 simulations.

Fig 2 shows the average proportion of policemen killed in the simulations for different combinations of *p* and *r* (each mean is calculated employing 480 simulations, averaging over different values of *n*). The white and light grey regions represent the cases in which a return to the status quo arises after the uprising: the number of policemen killed is less than 40%. In fact, these areas correspond to a high value for policemen’s precision and a low value for that of revolutionaries. As *r* increases or *p* decreases, the outcome of the simulations changes towards anarchy: these outcomes are represented by the darker grey areas, where the percentage of policemen killed is between 40% and 80%. Above a certain level for the two precision parameters, the situation changes from anarchy to successful revolution: the regions for successful revolutions, where the average percentage of policemen killed exceeds 80%, are coloured black.

An important feature of the figure is that the white and light grey areas are well below the 45 degree line: this means that policemen need a very high level of precision compared to that of revolutionaries in order to win the armed conflict. This is due to the revolutionaries’ strong advantage: in fact, they can hide among active citizens and attack when government forces are engaged in public order maintenance. This advantage results from the fact that policemen randomly draw one agent from the set of both active citizens and active revolutionaries within their vision radius (see rule P), and not from the set formed by revolutionaries only. This part of the model offers an incentive for revolutionaries to become active only when participation in spontaneous riots exceeds a minimum threshold. It also helps explain why, in the past, revolutionary movements occurred following strikes, protests and riots.

**Fig 3:**
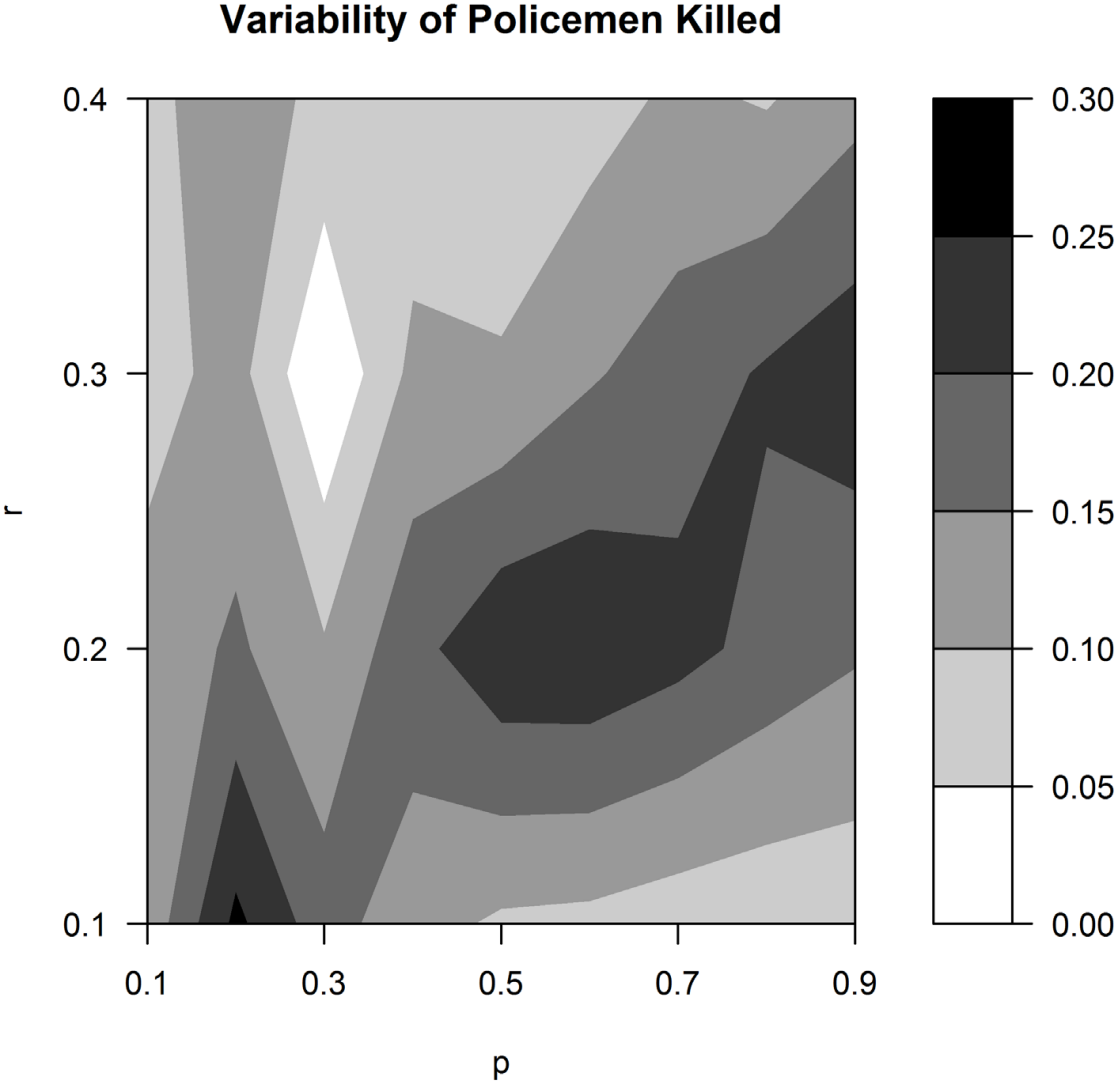
Variability of the proportion of policemen killed for different values of the two precision parameters. For each combination of *p* and *r*, the standard deviation of the proportion of policemen killed is calculated employing 480 simulations.

Fig 3 shows the same graph, albeit with the standard deviation of the proportion of policemen killed rather than the mean. First, it is interesting to note that areas characterised by anarchy, in average terms, are also associated with a high variability of policemen killed (the dark grey and black areas). In contrast, the regions corresponding to a return to the status quo and the regions of successful revolutions display much lower levels of volatility: this means that, in these areas, the same outcome is often observed, while in the regions where anarchy, on average, is observed it is easier to observe diverse outcomes.

One of the main features shared by many revolutions in history is that they were not anticipated, neither by the government nor by the opposition. This pattern was first observed by Kuran [6–8] in the dynamics of the French, Russian and Iranian revolutions and in the fall of communist regimes in Eastern Europe. A related interpretation was provided recently by Taleb and Treverton [30], who point out that apparently stable regimes may be less well equipped to manage political instability than countries that are often affected by disorder and turmoil, which leads to their decline in the presence of significant and unanticipated shocks.

**Fig 4:**
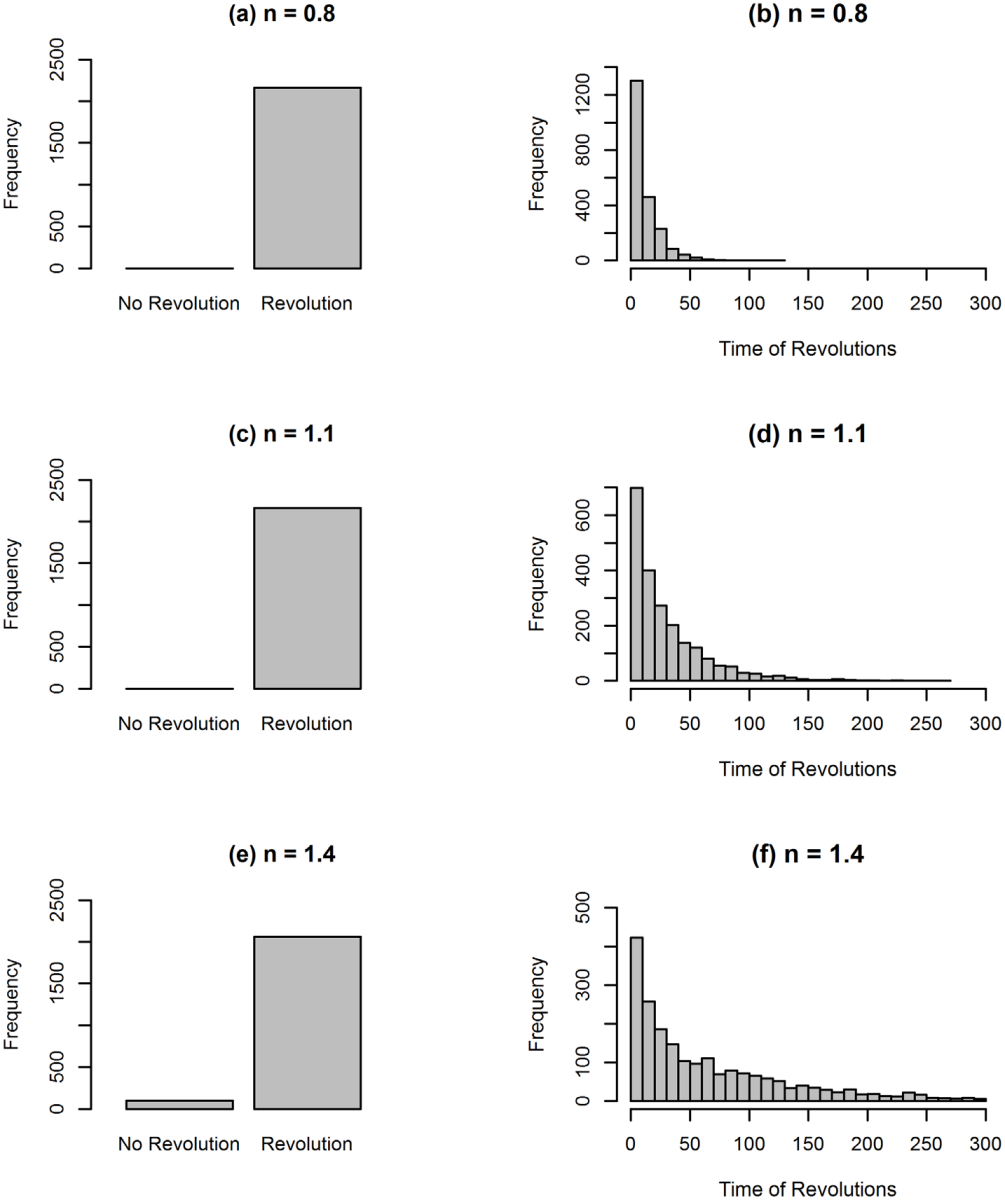
The unpredictability of revolutions. The graphs show how many simulations result in the occurrence of a revolution and the distribution of the time when rebellion occurs: (a) histogram of the number of revolutions that take place in 2,160 simulations for *n* = 0.8; (b) histogram of the time when revolutions occur for *n* = 0.8; (c) histogram of the number of revolutions that take place in 2,160 simulations for *n* = 1.1; (d) histogram of the time when revolutions occur for *n* = 1.1; (e) histogram of the number of revolutions that take place in 2,160 simulations for *n* = 1.4; (f) histogram of the time when revolutions occur for *n* = 1.4.

The model presented in this paper is able to explain the unpredictable nature of revolutions. In fact, Fig 4 shows for three different values of *n* (*n* = 0.8, *n* = 1.1, *n* = 1.4) how many of the 2,160 simulations result in a revolution and the distribution of the time when rebellion occurs (the simulations employed have different values of *p* and *r*, but these parameters do not affect the timing of revolutions). For low and medium values of *n*, revolutionaries become active in every simulation and the time of activation is concentrated within 50 time steps. By increasing the value of the revolutionaries’ threshold, a larger number of simulations do not result in a revolution, because the number of active citizens never reaches the level required for revolutionaries to become active, and the distribution of the time when rebellion occurs is more widespread. The revolutions generated by the model are therefore random events. In fact, it is impossible to anticipate if and when there will be a riot involving enough active citizens to activate the revolutionaries and generate an uprising. This behaviour of the model mimics real revolutionary events in which, as stressed by Goldstone [31] in the context of the Arab Spring, opposition elites or defected military officers and most individuals who want to rebel against the government have an incentive to hide their true feelings until the crucial moment arises. It is also impossible to know which episode will lead to mass, rather than local, mobilisation.

### Statistical Analysis

Using the same simulated data described in the previous subsection, several statistical models are estimated so as to help understand how the three outcomes of the model depend on the two precision parameters and the revolutionaries’ intervention threshold.

In each simulation *s* a binomial distribution is assumed for the number of policemen killed *z*_*s*_. The two parameters of this distribution are the number of policemen *P* and the probability of killing a policeman *k*, respectively. This last quantity, which can also be interpreted as a measure of the probability of a successful revolution, is assumed to be a function of the three most important parameters of the model, i.e. the precision of policemen *p*_*s*_, the precision of revolutionaries *r*_*s*_, the revolutionaries’ activation threshold *n*_*s*_. The probability function of the number of policemen killed is therefore:

d(zs)=Pzsk(ps,rs,ns)zs1-k(ps,rs,ns)P-zs,zs=0,1,⋯,P (7)

The model is estimated with different link functions for probability *k*, and the effect of *n* is included with a third-degree polynomial:

$$\begin{array}{c} k\left(p_{s},r_{s},n_{s}\right)=g\left(\beta_{0}+\beta_{1}p_{s}+\beta_{2}r_{s}+\beta_{3}n_{s}+\beta_{4}n_{s}^{2}+\beta_{5}n_{s}^{3}\right) \end{array}$$ (8)

where *g*(.) can be the linear, logit, probit or complementary log-log link function.

The results are presented in Table 2: in the first column, the linear probability model (LPM) is estimated using the ordinary least squares estimator; in the other three columns, a different generalised linear model is estimated using the maximum likelihood estimator, assuming the link function of the logit, probit and complementary log-log model, respectively.

**Table 2:** Estimates of the statistical models.

	LPM^a^	Logit^b^	Probit^c^	CLogLog^d^
*p*	−0.498**	−4.969**	−2.740**	−2.438**
	(0.006)	(0.014)	(0.008)	(0.007)
*r*	2.015**	20.120**	10.868**	9.154**
	(0.014)	(0.044)	(0.022)	(0.020)
*n*	−0.079	−0.854	−1.917**	−2.722**
	(0.582)	(1.145)	(0.641)	(0.601)
*n*^2^	0.381	3.950**	3.870**	5.093**
	(0.572)	(1.116)	(0.625)	(0.585)
*n*^3^	−0.211	−2.181**	−1.846**	−2.440**
	(0.183)	(0.354)	(0.198)	(0.185)
Constant	0.432*	−0.940*	−0.047	−0.118
	(0.193)	(0.382)	(0.214)	(0.200)
Observations	17,280	17,280	17,280	17,280
*R*^2^/pseudo-*R*^2^	0.623	0.692	0.685	0.656

^a^ Linear probability model estimated using the ordinary least squares estimator, standard errors corrected for heteroskedasticity.

^b^ Logit model estimated using the maximum likelihood estimator.

^c^ Probit model estimated using the maximum likelihood estimator.

^d^ Complementary log-log model estimated using the maximum likelihood estimator.

** Significant at 1%.

* Significant at 5%.

In all models, the two precision parameters have the expected signs: the policemen’s precision has a significant and negative impact on the probability of killing a policeman because the higher the precision of governmental forces, the larger the number of revolutionaries killed and, consequently, the lower the effectiveness of revolutionaries in killing policemen; on the other hand, as expected, the revolutionaries’ precision has a significant and positive effect on the probability of killing a policeman.

**Fig 5:**
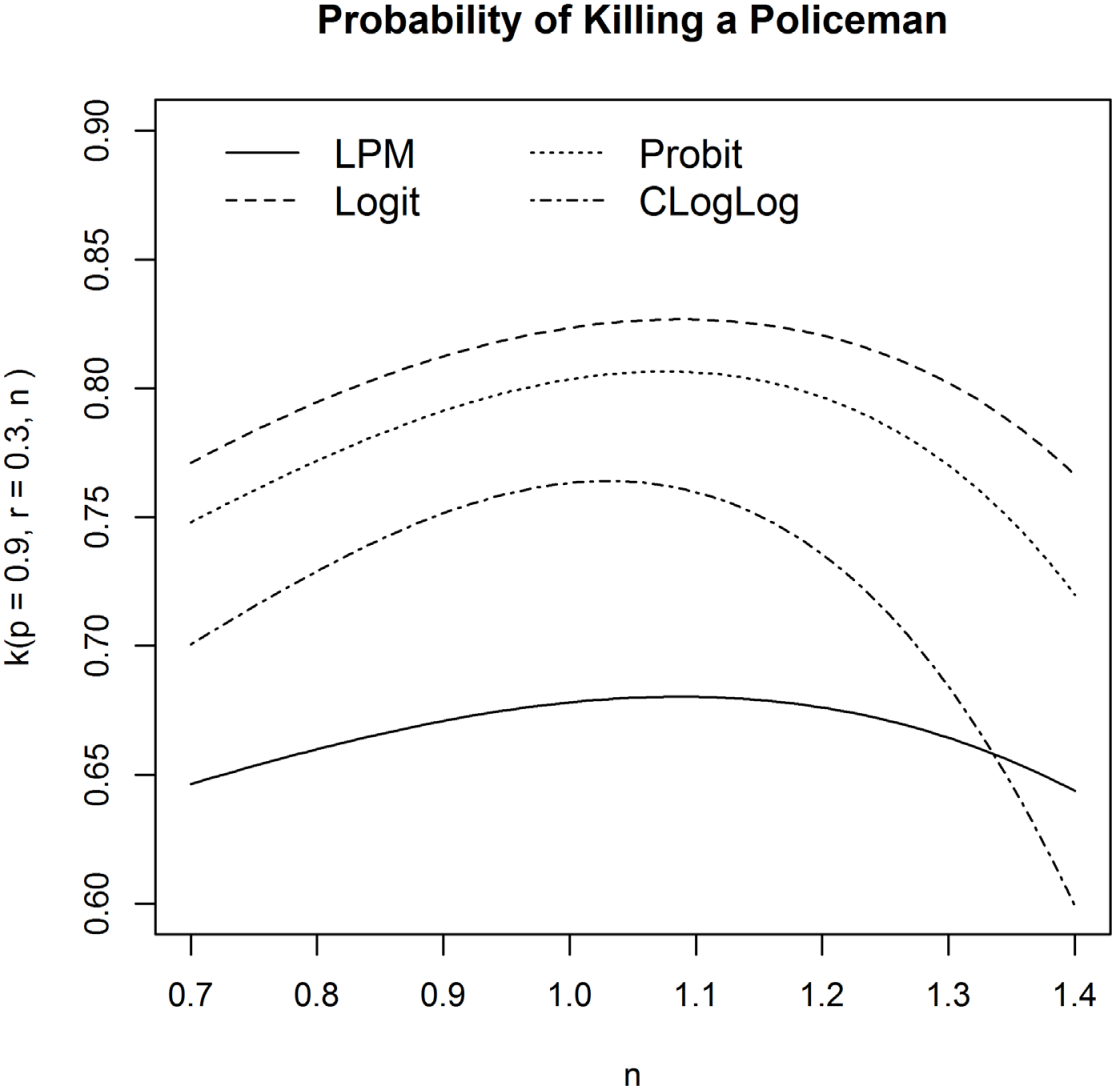
Probability of killing a policeman as a function of the revolutionaries’ threshold. Function *k*(0.9, 0.3, *n*) is plotted for the linear, logit, probit and complementary log-log model.

In order to analyse the effect of the revolutionaries’ threshold *n* on the probability of killing a policeman, function *k*(0.9, 0.3, *n*) is plotted in Fig 5 (*p* = 0.9 and *r* = 0.3 are plausible values for the two precision parameters) for the linear, logit, probit and complementary log-log model. In all specifications, it is evident that the probability of killing a policeman slightly increases in *n* up to a given value; then, the probability decreases markedly if *n* increases. A third-degree polynomial is preferred to a second-degree one because it allows this asymmetry to be captured. The intuition behind this shape is the following: for a revolutionary organisation, it is not optimal to start a revolution too early, when popular riots are small-scaled (small value of *n*), because it would easily come under fire from policemen. At the same time, however, revolutions may not occur if the revolutionary organisation waits too long (high value of *n*). According to these estimates, if the revolutionary organisation’s objective is to maximise the probability of a successful revolution, the optimal behaviour is to choose *n* in [1.0, 1.1], which suggests starting the uprising when the number of active agents is equal to that of governmental forces or exceeds it by about 10%.

## Discussion

Although the agent-based model presented in this paper is simple and makes no claim of incorporating all of the complex aspects involved in historical revolutions, it nevertheless captures several relevant stylised facts that are common to most revolutionary episodes in the real world.

The most important of these facts is represented by the multiplicity of different scenarios that can arise from a rebellion, i.e. a successful revolution, an anarchic scenario and the return to the status quo. The relevance of this last aspect can be understood by considering recent experience in the Arab Spring, where many rebellions, that seemed to start in 2011 in a similar way, resulted in completely different political outcomes.

Moreover, this model highlights a plausible dynamics, coherent with major political revolutions, that can be summarised as follows: a pre-revolutionary period characterised by spontaneous riots motivated mainly by poor economic conditions and social inequality, followed by a proper revolutionary rebellion where organised and politically oriented elements mobilise popular masses against the central authority. This dynamics mimics the sequence of events of most historical revolutions, and is consistent with the political science literature, which stresses the role played by revolutionary elites in the organisation of successful revolutions.

Furthermore, this paper examines the trade-off that revolutionaries face in deciding when to become active: if they start an uprising too early, when popular riots are minor, they will directly come under fire from policemen; on the other hand, if they wait too long, the revolution may not occur at all. If the revolutionary organisation’s objective is to maximise the probability of a successful revolution, the optimal threshold should balance these two opposite forces, and riots that do not exceed this minimum level of rebels will not degenerate into revolutions.

This paper also stresses the random nature of revolutions, pointing out that rebellions arise from interactions between many agents, determining their unpredictability: it is impossible to predict with certainty when and which riot will degenerate into a revolution. This consideration implies that similar countries, in terms of institutions and political systems, may experience revolutionary events at different points in time, or that some may not experience revolutions at all.

A policy implication that can be derived from the model is as follows. Let us suppose that a foreign state wants to intervene in another state to support a revolutionary group by providing more effective weapons in order to overthrow the existing government. In the framework of the model, this is translated into an increase in the revolutionaries’ effectiveness captured by parameter *r*. It is also assumed that, without external intervention, the initial configuration of precision parameters would have led to a rebellion followed by a return to the status quo. If the increase in revolutionaries’ precision is not sufficiently large, as shown in the graph in Fig 2, the political situation may degenerate from a relatively stable situation, the return to the status quo (the white and light grey areas in the figure), to an unstable one, characterised by a rebellion resulting in anarchy (the darker grey area in the figure). This implies that the foreign government should provide enough support in order to deliver a successful revolution as the final result (the black area in the figure). Mistakes in the calibration of this support may precipitate a country towards a state of persistent turmoil and civil war.

## Supporting Information

S1 Code The code that implements the model described in the paper. The model was written using NetLogo. The file allows users to change the parameter values and visualise the results both in the bidimensional space and the time series graphs. The simulated data employed in the analysis were generated using the BehaviorSpace tool in NetLogo; the statistical analysis was performed using R.
(NLOGO)

S1 Video The video of a successful revolution. The green circles represent quiet citizens, the orange squares are revolutionaries and the blue triangles are policemen. When a circle turns red, it means that the citizen has decided to become active; a black circle implies that the agent has been arrested. This video shows a revolution where all policemen are killed by revolutionaries: in fact, after the massive activation of citizens, the blue triangles disappear.
(MOV)

S2 Video The video of an anarchic scenario. This video shows a rebellion where all revolutionaries are killed by policemen, but where a high proportion of policemen also die: this implies that, even after the uprising, there is a persistent level of rebellion activity represented by the numerous red circles.
(MOV)

S3 Video The video of a failed revolution. This video shows a rebellion where all revolutionaries are killed by policemen and very few policemen die: this means that, after the uprising, there are very few red circles, representing active citizens, which immediately turn black because they are arrested by policemen.
(MOV)

S1 Dataset This file contains all of the simulations performed in this paper. Data are organised as a matrix in which each row corresponds to a simulation and each column represents a variable. In particular, the first column, called id, reports a progressive number that identifies each simulation. The other columns report the precision of policemen (*p*), the precision of revolutionaries (*r*), the activation threshold (*n*), the number of policemen who survive 300 time steps (called policemen), the number of revolutionaries who survive 300 time steps (called revolutionaries), and the time when the revolution occurs (called *t*_*rev*_). If *t*_*rev*_ assumes a value equal to 999, it means that no revolution occurs within 300 time steps.
(TXT)
